# Leiomyoma as a rare cause of small bowel obstruction: A case report

**DOI:** 10.1016/j.ijscr.2023.108473

**Published:** 2023-07-07

**Authors:** Husam Matalqah, Razan Yaseen, Maher Al-Hajjaj, Lina Matalkeh, Mohammad Matalkeh, Basil Al Taani

**Affiliations:** a King Abdullah University Hospital, Irbid, Jordan; b Department of Surgery, Aleppo University hospital, University of Aleppo, Aleppo, Syria; c Ministry of Health, Irbid, Jordan; d Jordan University of Science and Technology, Irbid, Jordan

**Keywords:** Small bowel obstruction, Leiomyoma, Case report

## Abstract

**Introduction and importance:**

Small bowel obstruction (SBO) is common in daily practice in emergency department. It has many causes such as adhesions or hernias. Other causes are obstruction by a bulky mass such as tumors of the intestine.

**Case presentation:**

We present a rare case of small bowel obstruction in a 48 years old male patient with Crohn's disease. Laparotomy showed a mass that caused the obstruction. Pathology report demonstrated that it is leiomyoma. This finding may cause a delay in making the diagnosis of SBO.

**Clinical discussion:**

Small bowel obstruction is considered an emergency case in the emergency. It needs special attention and advanced medical practice to reach the diagnosis. Rare cases may lead to delay the diagnosis because they present unusually.

**Conclusion:**

Physicians should consider leiomyoma as a cause of SBO with patients without clear cause of SBO.

## Highlights

• Small bowel obstruction (SBO) is a condition with many symptoms, including constipation, vomiting, and abdominal distension.
• Leiomyoma’s (fibroids) are benign smooth muscle tumors of the uterine myometrium.
• Small bowel obstruction with leiomyoma is considered a rare entity and drain special attention.
• Accurate monitoring and imaging can serve to reach the definitive diagnosis and avoid bowel ischemia, perforation, peritonitis, and sepsis.

## 1 Introduction

Small bowel obstruction is one of the common surgical emergencies we see in our day-to-day practice. Postoperative adhesions (60 %) are the most common cause of small bowel obstruction, followed by malignant deposits or extrinsic compression of the small bowel by other primary malignancies (20 %) and intestinal hernias (10 %) [1].

Intraluminal causes of small bowel obstruction in adults are rare.

Emergent surgery is necessary for patients with clinical or radiological signs suggestive of bowel ischemia. Even with the advent of laparoscopic surgery, intra-abdominal adhesions remain a significant cause of SBO, accounting for 65 % of cases, among other etiologies such as hernias, neoplasms, and Crohn's disease. Adhesive SBO (ASBO) contributes substantial financial burden, need for hospitalization, time away from routine life activities, and psychological impact [2].

Here, we report a rare case of small intestinal obstruction caused by submucosal leiomyoma in a patient with previously diagnosed with Crohn's disease.

We declare that our work has been reported in line with the SCARE criteria [3].

## 2 Case presentation

A 48-year-old male patient presented with three days of progressive generalized abdominal pain, bloating, and weight loss. He was diagnosed in another hospital with Crohn's disease 25 years previously, which was controlled poorly. Vital signs were as follow: blood pressure was 113/60 mm Hg, pulse was 98/min, temperature was 38.1 C0, and respiratory rate was 22/min.

**Fig. 1:**
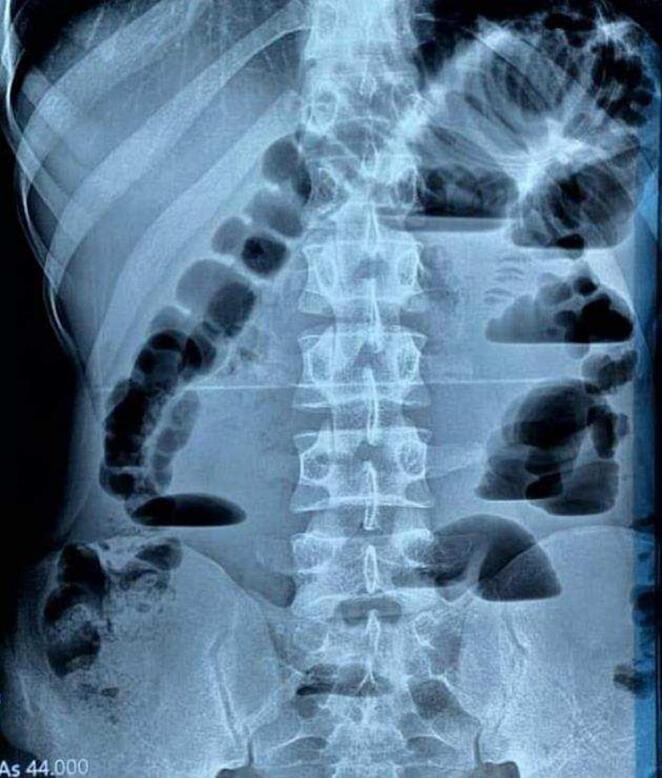
Abdominal X-ray showing loop dilation of small bowel.

**Fig. 2:**
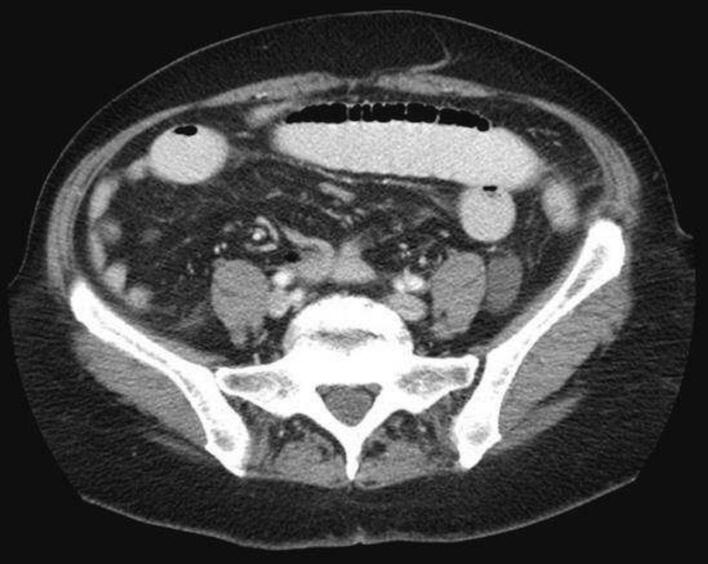
Computed tomography of abdomen and pelvis with contrast showing dilated loops with air-fluid levels.

On physical examination, there were abdominal tenderness and distension without rebound or guarding. There were no hernias or abdominal scars. Other systems were normal. Laboratory findings on admission are shown in Table 1. He had leukocytosis (14 × 105) with mild anemia (9.7 mg/dl). We performed an upright abdominal X-ray (Fig. 1) which showed dilated loops of small bowel with air-fluid levels. Computed tomography of the abdomen and pelvis with contrast showed dilated loops with air-fluid levels (Fig. 2). Based on clinical and radiological findings, the patient was diagnosed with small bowel obstruction (SBO).

**Table 1:** Laboratories at presentation.

Wight blood cell count	Hemoglobin	Platelets	Creatinine	CRP	Urea	Glucose	Na+	K+
14 × 10^5^/ml	9.7 × 10^5^ g/dl	300 × 10^5^/mcl	1 mg/dl	76	26 mg/dl	85 mg/dl	142 mEq/L	4.9 mEq/L

We started with intravenous antibiotics and resuscitation with normal saline. We inserted a nasogastric tube for bowel relief. We monitored vital signs hourly.

On the next day of admission, we decided to perform an exploratory laparotomy under general anesthesia because of the increase in abdominal pain.

Laparotomy was performed, through a mid-line incision. There was minimal intraperitoneal serous fluid and 25 cm. Proximal to the mid portion of the jejunum; we found a severely dilated loop with a palpable mass after the dilation. We resected this segment containing the mass and it was sent to the pathologist. Stapled anastomosis was carried out. We close the incision with 2-0 simple nylon sutures.

Histopathology showed 2.5 × 3 cm submucosal leiomyoma (Fig. 2). The course after surgery was uneventful. He passed flatus on day 2 after surgery.

On day 5 after surgery, we discharged him with no complaints. He presented to the out-patient clinic for follow-up after 13 days of discharge. He had no symptoms, and he could eat more food with good defecation. His wound showed healing well, so we removed the sutures. We sent him to complete his treatment of Crohn's disease with his gastroenterologist.

**Fig. 3:**
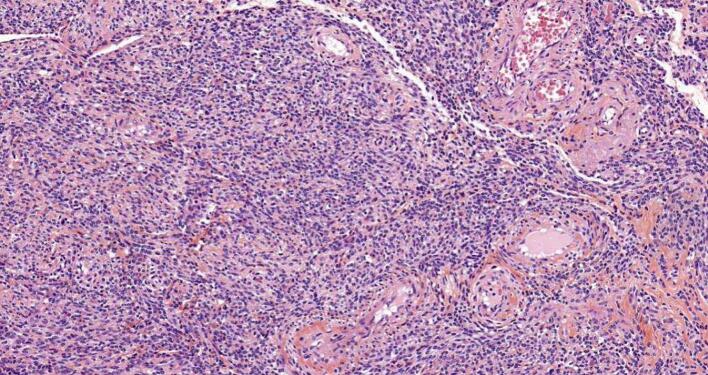
Histology slide from the intestine showing submucosal leiomyoma on heamtoxylin and eosin stain.

In addition, he was advised to stop smoking completely, reduce the use of NSAIDs, and increase the intake of fibers in his meals (Fig. 3).

## 3 Discussion

While SBOs are a known complication of Crohn's disease, these blockages are usually the result of inflammatory strictures or fistulas that narrow the intestinal lumen. However, several case reports have described obstructions resulting from intraluminal tumors in patients with Crohn's disease, with adenocarcinomas being the most common [4].

Leiomyomas are benign tumors that originate from smooth muscle cells derived from mesenchymal tissue. Although they are most commonly found in the uterus, leiomyomas can occur wherever smooth muscle is present, including along the entire length of the intestine. Within the small bowel, leiomyomas are predominantly found in the jejunum [5].

While no direct links have been made in the literature between Crohn's disease and leiomyoma formation, Crohn's has been identified as a risk factor for the formation of adenocarcinomas, which have a similar potential to obstruct the intestinal lumen [[6], [7]].

Our case was different. We had a rare cause of small bowel obstruction. It was a leiomyoma that led to SBO in a male patient with Crohn's disease. Conservative treatment did not success. We performed laparotomy to relieve the obstruction and reach the definitive diagnosis.

## 4 Conclusion

This case is considered very rare and it needs special consideration of physicians. SBO have many common causes. Nevertheless, there are rare causes that make the diagnosis too difficult to make. Leiomyoma is one of these rare causes.

## Consent

Written informed consent was obtained from the patient for publication of this case report and accompanying images. A copy of the written consent is available for review by the Editor-in-Chief of this journal on request.

## Ethical approval

As this publication is a case report that contains no identifiable content to the patient, this publication was exempt from ethical approval by the Human Research Protection Program (HRPP) and its Institutional Review Board (IRB) at the Aleppo University Hospital, Aleppo, Syria.

## Funding

We declare that we didn't require any funding.

## Author contribution

Husam Matalaqah, Razan Yassen, Lina Matalkeh, Mohammad Matalkeh, and Basil Al Taani: writing the case report.

Maher Al-Hajjaj: Data collection and Writing-Original draft preparation.

## Guarantor

Maher Al-Hajjaj

## Research registration number

Not applicable.

## Declaration of competing interest

The authors declare that they have no conflict of interest.

## References

[1] Tito W.A., Sarr M.G., Zuidema G.D., Nyhus L.M. (1996). Intestinal Obstructions. Schakelford’s Surgery of the Alimentary Tract.

[2] Tong J.W.V., Lingam P., Shelat V.G. (2020). Adhesive small bowel obstruction–an update. Acute Medicine & Surgery.

[3] Agha R.A., Franchi T., Sohrab C., Mathew G., Kirwan A., Thomas A. (2020). The SCARE 2020 guideline: updating consensus Surgical Case Report (SCARE) guidelines. Int. J. Surg..

[4] Shah J., Etienne D., Reddy M., Kothadia J.P., Shahidullah A., Baqui A.A.M.A. (2018). Crohn’s disease manifesting as a duodenal obstruction: an unusual case. Gastroenterol. Res..

[5] Blanchard D.K., Budde J.M., Hatch G.F. (2000). Tumors of the small intestine. World J. Surg..

[6] van Schaik F.D.M., Mooiweer E., van der Have M. (2013). Adenomas in patients with inflammatory bowel disease are associated with an increased risk of advanced neoplasia. Inflamm. Bowel Dis..

[7] Solem C.A., Harmsen W.S., Zinsmeister A.R., Loftus E.V. (2004). Small intestinal adenocarcinoma in Crohn’s disease: a case-control study. Inflamm. Bowel Dis..

